# Effect of Violet-Blue Light on *Streptococcus mutans*-Induced Enamel Demineralization

**DOI:** 10.3390/dj6020006

**Published:** 2018-03-21

**Authors:** Grace Gomez Felix Gomez, Frank Lippert, Masatoshi Ando, Andrea Ferreira Zandona, George J. Eckert, Richard L. Gregory

**Affiliations:** 1 Department of Biomedical and Applied Sciences, Indiana University School of Dentistry, Indianapolis, IN 46202, USA; 2 Department of Cariology, Operative Dentistry and Dental Public Health, Indiana University School of Dentistry, Indianapolis, IN 46202, USA; flippert@iu.edu (F.L.); mando@iu.edu (M.A.); 3 Department of Operative Dentistry, University of North Carolina, Chapel Hill, NC 27599, USA; azandona@email.unc.edu; 4 Department of Biostatistics, Indiana University, Indianapolis, IN 46202, USA; geckert@iu.edu

**Keywords:** violet-blue light, phototherapy, *Streptococcus mutans*, dental caries

## Abstract

Background: This in vitro study determined the effectiveness of violet-blue light (405 nm) on inhibiting *Streptococcus mutans*-induced enamel demineralization. Materials and Methods: *S. mutans* UA159 biofilm was grown on human enamel specimens for 13 h in 5% CO_2_ at 37 °C with/without 1% sucrose. Wet biofilm was treated twice daily with violet-blue light for five minutes over five days. A six-hour reincubation was included daily between treatments excluding the final day. Biofilms were harvested and colony forming units (CFU) were quantitated. Lesion depth (*L*) and mineral loss (∆*Z*) were quantified using transverse microradiography (TMR). Quantitative light-induced fluorescence Biluminator (QLF-D) was used to determine mean fluorescence loss. Data were analyzed using one-way analysis of variance (ANOVA) to compare differences in means. Results: The results demonstrated a significant reduction in CFUs between treated and non-treated groups grown with/without 1% sucrose. ∆*Z* was significantly reduced for specimens exposed to biofilms grown without sucrose with violet-blue light. There was only a trend on reduction of ∆*Z* with sucrose and with *L* on both groups. There were no differences in fluorescence-derived parameters between the groups. Conclusions: Within the limitations of the study, the results indicate that violet-blue light can serve as an adjunct prophylactic treatment for reducing *S. mutans* biofilm formation and enamel mineral loss.

## 1. Introduction

Dental caries is a biofilm-mediated disease; therefore, biofilm is indispensable for caries initiation. There are a multitude of factors involved in the initiation and progression of caries [1]. Dental caries is preventable by controlling a few of the many factors involved in the development of the disease [2]. Since disease management is more effective in the early stages, the dogma has been to detect carious lesions at its initial stage to prevent its progression to cavitation. Currently, various preventive strategies ranging from natural products to nanotechnological approaches are under development focusing on biofilm modulation. One novel method is phototherapy to inactivate oral biofilm formation [3]. *S. mutans* is considered a primary cariogenic bacterium and has the capacity to form biofilm, produce and resist acidic conditions [4].

Light therapy studies have been applied for several oral microorganisms [5]. *S. mutans* had been studied widely with photodynamic therapy using exogenous photosensitizers such as Erythrosine, Rose Bengal, Toluidine Blue, and Malachite Green, among others [6,7,8,9]. Few studies have focused on phototherapy without the presence of exogenous photosensitizers [10,11,12,13]. Photosensitizers or photoactivable compounds can be either added exogenously, or present endogenous, within the bacterium. These photosensitizers absorb light of a specific wavelength or a range of wavelengths, get activated, and undergo a transition of energy from a ground state to an excited singlet state. Subsequently the transfer of energy from the excited photosensitizer with the available molecular oxygen produces reactive oxygen species (ROS), mediating bacterial destruction [5,6,7,8,9,10,11,12]. Preliminary studies have shown the photo inhibitory effects of violet-blue light on *S. mutans* biofilm [14]. The aim of this in vitro study was to determine the effect of violet-blue light at the surface level of the tooth, namely enamel, and on the *S. mutans* biofilm.

## 2. Materials and Methods

### 2.1. Study Design

**Figure 1:**
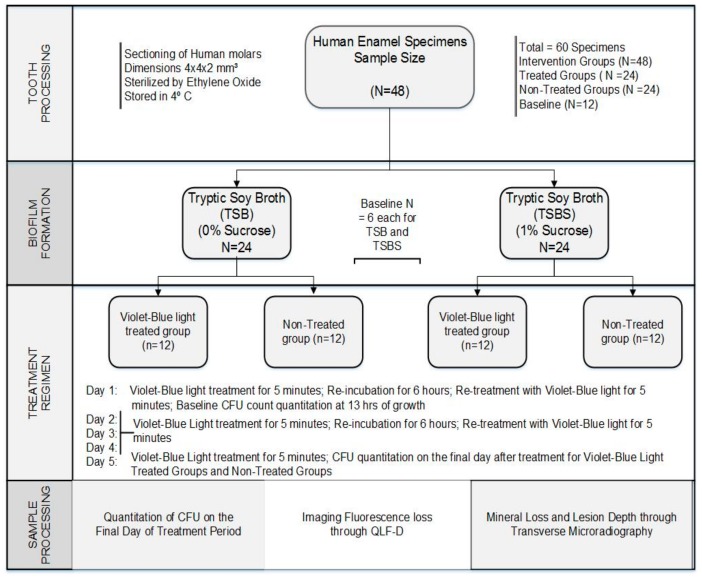
Flowchart of the study design.

Sixty enamel specimens were used in this experiment. From this sample pool, 48 specimens were randomly selected for randomization into 4 intervention groups that included violet-blue light treated and non-treated control groups. Group 1 (*n* = 12) consisted of biofilms grown with Tryptic Soy Broth (TSB) with 0% sucrose and treated with violet-blue light; Group 2 (*n* = 12) consisted of biofilms grown in TSBS with 1% sucrose and treated with violet-blue light; Group 3 (*n* = 12) consisted of biofilms grown with TSB not treated with violet-blue light; and Group 4 (*n* = 12) consisted biofilms grown with TSBS not treated with violet-blue light. Analysis of specimens from the intervention group was done at the end of a 5-day treatment period [13]. The remaining 12 specimens were used for baseline measurements at the end of 13 h period before the intervention. Baseline samples (*n* = 6 each) for both TSB and TBSS were used. Baseline measurements before treatment provide information on the actual effect of the treatment. Groups 1 and 2 were treated with violet-blue light (13 mW/cm^2^; ~4 J/cm^2^) for 5 min, 2 times a day, 4 days a week; but only once on the 5th day of the treatment. Quantification of biofilm cells was performed to obtain a baseline measurement at 13 h and on the final day of the treatment period. Additionally, all 60 of the enamel specimens were imaged for fluorescence loss using QLF-D and sectioned to determine mineral loss and lesion depth through TMR (Figure 1).

### 2.2. Bacterial Culture Conditions

*S. mutans* (UA159, serotype c strain) stored at −80 °C with glycerol was used in this study. The bacteria were cultured on mitis salivaris sucrose bacitracin (MSSB, Anaerobe Systems, Morgan Hill, CA, USA) agar plates. *S. mutans* was grown in 5 mL of Tryptic Soy Broth (TSB, Acumedia, Baltimore, MD, USA) for 24 h in a 5% CO_2_ incubator at 37 °C [15].

### 2.3. Enamel Specimen Preparation

Sixty enamel specimens were prepared from extracted human molars without any cracks, fractures or caries (Institutional Review Board (IRB) approval (# NS0911-07). A Lap Craft L’il Trimmer^TM^ (Powell, OH, USA) was used to decoronate the crown portion of the tooth. Enamel specimens with dimensions of 4 × 4 × 2 mm^3^ were cut using a Isomet saw (Buehler, Lake Bluff, IL, USA). Specimens were ground sequentially with 500, 1200, 2400 and 4000 grit silicon carbide paper with a RotoPol-31/RotoForce-4polishing/grinding machine (Struers, Cleveland, OH, USA). Specimens were ground with each sandpaper grit for 4 s and the specimen thickness was reduced to 2 mm. The actual thickness ranged from 1.6 to 2.1 mm. Clear nail varnish was used to coat all sides and the bottom of the enamel specimens. Approximately one third of the top surface of the enamel specimens was covered with nail varnish. Quality assurance of the specimens was done with a microscope at 20× magnification to determine absence of cracks, fractures or fissures. Compromised specimens were excluded [16] (modified from Lippert and Juthani, 2015).

### 2.4. Specimen Sterilization

Human enamel specimens were rinsed for 3 min, sonicated for 3 min, and again rinsed for 3 min with deionized water. The specimens were placed in moist cotton gauze, sealed in a whirl pak bag (Sigma-Aldrich, St. Louis, MO, USA), and sterilized with ethylene oxide gas [17].

### 2.5. Biofilm Formation

Overnight bacterial broth cultures were diluted 1:100 with TSB or TSBS. *S. mutans* biofilm cells were grown in sterile 96-well microtiter plates (Fisher Scientific, Co., Newark, DE, USA) with (*n* = 12) and without (*n* = 12) enamel specimens [18]. The biofilm cells were incubated for 13 h in a 5% CO_2_ incubator at 37 °C to reach the logarithmic phase of growth. A gap of one well was left in between the biofilm samples and a 2-well distance was maintained between TSB and TSBS groups.

### 2.6. Violet-Blue LIGHT source

The light emitted from the quantitative light-induced fluorescence (QLF-clin, Inspektor Research Systems^TM^ BV, Amsterdam, The Netherlands) was used as a light source for the treatment of *S. mutans* biofilm cells in this study. It employs a 35-Watt Xenon arc lamp, and violet-blue light is filtered through a high-pass band filter. The spectral range of the violet-blue light is within a range of 380 to 450 nm with a peak wavelength at 405 nm [14].

### 2.7. Treatment with Violet-Blue Light

Before irradiation, planktonic or supernatant liquid was removed from above the biofilm cells and the wet biofilm was exposed to violet-blue light continuously for 5 min. The pH of each of the supernatants were measured. The distance between the bottom of the microtiter plate and the light source was kept constant at 2.0 cm. A black background was used to avoid scattering of light. Seal mate was placed as a barrier between the sample and the light source opening. Immediately after exposure, freshly prepared TSB or TSBS growth medium was added to each well. The treated biofilm cells were reincubated for 6 h in a 5% CO_2_ incubator at 37 °C [14]. After 6 h, the biofilm cells were again treated with violet-blue light for 5 min and reincubated with fresh TSB or TSBS for 13 h, until the next treatment on the following consecutive day. The procedure was repeated for 5 days, except on the final day, when only one treatment was provided without the 6 h reincubation.

### 2.8. Quantification of Colony-Forming Units

At the end of fifth day of the experiment, supernatant liquid was removed for pH measurements. Baseline CFUs of the 13 h biofilm with TSB and TSBS on the first day before the treatment were also obtained. CFUs of violet-blue light treated and non-treated groups in TSB and TSBS were obtained from the final day of the treatment regimen. Biofilm at the bottom of the plate was gently washed once with 0.9% saline. Enamel specimens were carefully removed from the microtiter plate and placed in 1 mL of saline solution in mini centrifuge tubes, vortexed for 10 s, sonicated on ice for 20 s, and again vortexed for 10 s [14,19]. Serial dilutions of the bacterial samples were prepared with 0.9% saline and plated in duplicates on Tryptic Soy Agar (TSA) plates using a spiral plater (Spiral System^TM,^ Cincinnati, OH, USA). TSA agar plates were incubated for 48 h at 37 °C in a 5% CO_2_ incubator, and the colony-forming units (CFUs) were counted by an automated colony counter using Protocol^TM^ (Synbiosis Inc., Frederick, MD, USA) software.

### 2.9. Quantitative Light-Induced Fluorescence

A quantitative light-induced fluorescence biluminator (QLF-D) was used to acquire images of the enamel specimens. A jig was prepared to secure the enamel specimen with silicone rubber (Oomo-30). The images were acquired through an illumination tube fitted on a SLR camera with white and blue light-emitting diodes (LED) under dark conditions. Fluorescence images of enamel specimens were obtained using a C3 proprietary software on QLF-D. The images were digitally archived for further analysis of mineral loss or lesion depth through QA2 analysis software. The QLF-D parameters mean fluorescence loss, delta *F* (∆*F*); maximum fluorescence loss (Δ*F*_max_); lesion volume, delta *Q* (∆*Q*); and lesion area (Area) were collected [20].

### 2.10. Transverse Microradiography

Enamel specimens were treated briefly with 70% ethyl alcohol and stored under moistened conditions with deionized water (diH_2_O). Enamel specimens were superglued to acrylic rods and enamel sections of 100 ± 20 µm thickness were prepared with a hard tissue microtome. One section per specimen was selected to be imaged. The enamel sections were placed on an ultra-resolution flat plate sized 5 × 5 × 2 mm^3^ (Microchrome Technology Inc., San Jose, CA, USA). Calibration of the TMR PSL Imaging System (Thermo-Kevex PXS5-928WB-LV, Tube 48934, Photonic Science Limited, East Sussex, UK) with respect to its absorption coefficient was done with an aluminum step wedge for an acceptable correlation of 0.99970. Imaging of the enamel specimens were obtained through X-ray source with a 45 kV voltage and a current of 45 µA. The obtained images were read and processed using TMRD1 5.0.01 software and finally analyzed through TMR2006 software v.3.0.0.18 (Inspektor Research Systems BV, Amsterdam, The Netherlands). Mineral loss (∆*Z*) and lesion depth (*L*) were determined.

### 2.11. pH Measurements

A pH meter (Accumet, Fisher Scientific, Pittsburgh, PA, USA) was used to measure the pH of the pooled samples of every group. The supernatant containing planktonic cells on top of the biofilm was removed before light irradiation for pH measurement. The pH was measured on the first day before the treatment and on the fifth or final day of the treatment regimen.

### 2.12. Statistical Analysis

One-way ANOVA was performed for TSB and TSBS groups, followed by pairwise group comparisons for baseline, violet-blue light treated and non-treated groups, to determine any difference in CFU. QLF-D parameters such as fluorescence loss (∆*F*), lesion area (Area), lesion volume (∆*Q*) and lesion depth (*L*) and mineral loss (∆*Z*) were obtained through transverse microradiography (TMR).

## 3. Results

### 3.1. Photoinhibition of S. mutans Biofilm on Human Enamel Specimens

**Figure 2:**
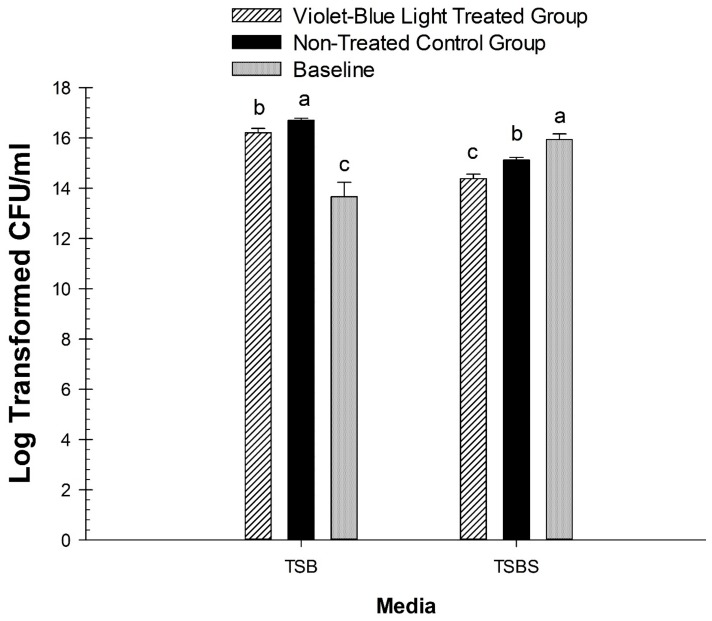
Comparison of baseline *S. mutans* biofilm CFU with treated and non-treated TSB and TSBS groups. The Log CFUs of the violet-blue light treated and non-treated group on the 5th day of the treatment were compared with the baseline counts at 13 h for TSB and TSBS. Different lower-case letters represent significant differences between groups, with comparisons performed separately within each media.

Baseline (*n* = 6) *S. mutans* biofilm grown in the absence of sucrose with TSB had statistically lower CFU numbers than the violet-blue light treated groups (*p* < 0.001) and also with non-treated groups (*p* < 0.0001). Baseline *S. mutans* biofilm grown in the presence of sucrose with TSBS had statistically higher CFU numbers than the violet-blue light treated groups (*p* < 0.0001) and the non-treated groups (*p* = 0.0149). Baseline CFU was obtained at 13 h for TSB and TSBS, and was compared against the CFU obtained on the final day of the intervention for the treated and non-treated groups within their respective media (Figure 2).

### 3.2. Effect of Violet-Blue Light on the Viability of S. mutans Biofilm Cells

The results of the photoinhibition of violet-blue light at the end of the treatment demonstrated that violet-blue light treated groups (*n* = 12) had significantly lower CFUs than the non-treated control groups (*n* = 12) with TSB (*p* = 0.0333) and similar significantly different results were obtained with TSBS (*p* = 0.0008). There was an approximately 28% reduction of bacterial numbers in TSB and 48% in TSBS (Figure 2).

### 3.3. Effect of Violet-Blue Light on the Lesion Depth by Fluorescence Image Analysis of Enamel Specimens through QLF-D

With respect to QLF-D parameters, there were no differences between the baseline value, violet-blue light treated and non-treated groups, (*p* = 0.37 for Δ*F*, *p* = 0.40 for Δ*F*_max_, *p* = 0.40 for Δ*Q*, *p* = 0.41 for lesion area, *p* = 0.12) on human enamel specimens with *S. mutans* biofilm with TSB (Table 1). Enamel specimens subjected to *S. mutans* biofilm in TSBS demonstrated that there were significant differences between the baseline and the violet-blue light treated groups and non-treated control groups. ∆*F*, ∆*F*_max_, ∆*Q*, Area were significantly lower for baseline values than the violet-blue light and non-treated control groups (*p* < 0.0001). The photoinhibitory effect of the violet-blue light treated and non-treated groups indicated that there were no significant differences in TSBS (*p* > 0.08) (Table 1).

### 3.4. Effect of Violet-Blue Light on Mineral Loss and Lesion Depth through Transverse Microradiography

**Figure 3:**
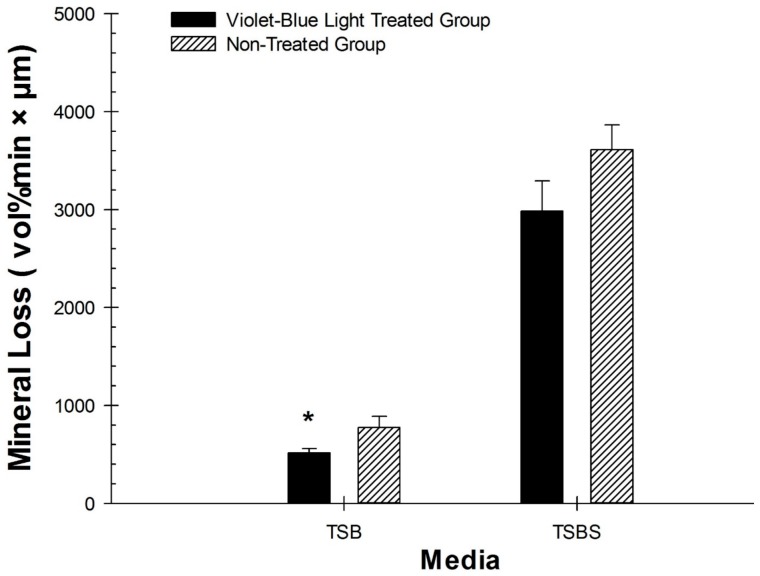
Comparison of the effect of violet-blue light on the mineral loss (TMR) produced by *S. mutans* biofilm on human enamel specimens in TSB and TSBS. Asterisks indicate statistical significance. Significance level was kept at *p* < 0.05.

The photoinibitory properties of violet-blue light was effective with *S. mutans* biofilm exhibiting less ∆Z (*p* = 0.0293) with TSB (Figure 3). However, there was no significant difference in ∆*Z* found in *S. mutans* biofilm formed with TSBS (*p* = 0.09) (Figure 3). Violet-blue light treated groups and non-treated control groups did not have significantly different lesion depths (*L*) in TSB or TSBS (*p* > 0.14). Baseline enamel specimens had less ∆*Z* and (*L*) compared to violet-blue light treated (*p* < 0.001) and non-treated control groups (*p* ≤ 0.0001).

### 3.5. pH Measurements

pH measurements of the supernatant or planktonic fluids at the beginning of the treatment on the first day were obtained. The violet-blue light treated group in TSB had a pH of 4.96 before treatment and the non-treated control groups had a pH of 4.99. The pH of planktonic or supernatant fluids in TSBS was 4.43 with violet-blue light treated groups and non-treated control groups had a pH of 4.45 on the first day before the treatment. On the final day of treatment, the pH values with TSB were 4.88 and 4.85 in the treated and in the non-treated groups, respectively. For TSBS, the pH values were 4.1 in both treated and non-treated groups.

## 4. Discussion

The current study demonstrated that violet-blue light had a statistically significant photoinhibitory effect on the number of CFUs of *S. mutans* after 5 days of treatment. Irrespective of the presence or absence of 1% sucrose, treatment with violet-blue light provided a reduction in the numbers of *S. mutans* biofilm cells. This effectiveness was based on a 13 h old biofilm, with two treatments on each of the first 4 days and one treatment on the final day of the treatment regimen. There was also a 6 h reincubation period in between the treatments with no reincubation on the final day of the treatment period. Previously we reported that metabolic activity of *S. mutans* biofilm at 0 h was reduced significantly compared to after 2 and 6 h of reincubation [21] (Gomez et al., *J. Oral Sci.*—in press). The baseline CFUs of *S. mutans* at 13 h were lower than the violet-blue light treated TSB and TSBS groups. These results demonstrated that, although violet-blue light was effective in reducing the numbers of the bacteria, there was regrowth of bacteria after photoinactivation. The findings correlate with the previous findings related to significant reduction in metabolic activity at 0 h compared to that activity following 2 and 6 h. It would be ideal to harvest bacterial cells after each time period to determine the temporal variation in bacterial viability with violet-blue light treatment. The supernatants containing planktonic bacteria above the biofilm were removed and used for pH measurements. Measuring the pH of biofilm would be an alternative option.

In relation to the QLF-D parameters, unlike the significant reduction of CFU numbers in the violet-blue light treated groups in TSB, there were no significant differences between the ∆*F*, ∆*F*_max_, ∆*Q*, Area. Though there was slight reduction in all the QLF-D parameters, it was not statistically different from the non-treated groups. The results indicate that violet-blue light may inactivate the bacteria in a biofilm environment. Since the QLF-D parameters were not much different between the baseline and experimental groups, variations of the enamel surface would also have contributed to this finding. Another possible explanation is that violet-blue light affects the architecture and development of the extra polysaccharide matrix of the biofilm. *S. mutans* utilizes sucrose to form extracellular polysaccharides (EPS) and enmesh bacterial cells together to form microcolonies with acidic pockets [22,23]. A recent report found that blue light did not have an effect on the soluble EPS but had an increased effect on insoluble EPS, which is cariogenic. They also found that CFUs for *S. mutans* biofilm cells treated with blue light were significantly reduced, compared to the negative control 0.89% NaCl. However, viability was significantly reduced with 0.12% Chlorhexidine compared to treatment with light [13]. In the present study, with TSBS there was no significant difference in QLF-D parameters between the violet-blue light treated and non-treated control groups. There was a slight reduction in all the QLF-D parameters mentioned above in the violet-blue light treated groups compared to non-treated groups, with minimal deviation, but this was not statistically significant. The short-term biofilm model with two replenishments of fresh media on the undisturbed biofilm on the enamel specimen would have contributed to balanced bacterial metabolism, with less degradation of EPS causing reduced enamel demineralization [24]. The baseline values had lower QLF-D parameters and were significantly different than the violet-blue and non-treated groups.

The gold standard method for determining lesion depth is to section enamel specimens, process microradiography images, and analyze them. The present findings supported that baseline values for mineral loss and lesion depth were significantly lower compared to violet-blue light treated groups and non-treated control groups. In the absence of sucrose with TSB, lesion depth and mineral loss were significantly lower compared to the non-treated groups, however this was not found with TSBS-grown groups. Early caries detection devices such as QLF differ in sensitivity based on enamel or dentin. It has lower sensitivity with enamel compared to dentin. Artifacts on the surface of enamel such as sucrose-grown biofilm may have contributed to the above findings and might be one of the limitations of the study. Future studies directed at simulating in vivo carious lesions induced by *S. mutans*, in addition to saliva and pellicle formation, would provide in-depth results.

## 5. Conclusions

Colony-forming units (CFU), and lesion depth, measured as mineral loss or fluorescence loss, measured through QLF-D confirmed by the gold standard of transverse microradiography were compared between violet-blue light treated and non-treated groups in both TSB and TSBS at the end of the treatment period. Violet-blue light had an inhibitory effect on the bacterial viability of *S. mutans* biofilm in both TSB and TSBS. There was no statistically significant effect of violet-blue light on the mineral level of the tooth surface or the mineral loss obtained through QLF-D. There was a slight reduction in the amount of loss of fluorescence. Mineral loss obtained through TMR was statistically significant in the violet-blue light treated group; however, lesion depth was not statically significant. Violet-blue light has a more effective photoinhibitory effect at the bacterial level on the surface of the tooth than at the mineral level.

## Figures and Tables

**Table 1:** Log transformation of QLF-D parameters on the effect of violet-blue light on the lesion depth by image analysis of loss of fluorescence on human enamel specimens by *S. mutans* biofilm grown in TSB or TSBS through QLF-D. *S. mutans* in TSB had no significant group differences for any of the QLF-D parameters. TSBS baseline had significantly lower QLF-D parameters than Blue (*p* < 0.001) and No Blue (*p* < 0.0001), while Blue and No Blue were not significantly different from one another (*p* > 0.08).

Log Transformed QLF-D Parameters	Media	Baseline		Violet-Blue Light		Non-Treated Light	
		*N*	Mean (SE)	*N*	Mean (SE)	*N*	Mean (SE)
delta *F*	TSB	6	0.61 (0.93)	12	1.50 (0.52)	12	1.86 (0.40)
	TSBS	6	0.52 (0.90)*	12	3.08 (0.17)	12	3.52 (0.09)
delta *F*_max_	TSB	6	0.91 (1.03)	12	1.90 (0.58)	12	2.25 (0.45)
	TSBS	6	0.75 (0.97)*	12	3.48 (0.15)	12	3.82 (0.07)
delta *Q*	TSB	6	4.52 (2.25)	12	7.16 (1.31)	12	7.50 (1.11)
	TSBS	6	4.23 (2.13)*	12	10.70 (0.28)	12	11.22 (0.28)
Area	TSB	6	3.16 (1.81)	12	5.29 (1.04)	12	5.46 (0.90)
	TSBS	6	2.95 (1.72)*	12	7.62 (0.12)	12	7.70 (0.20)

Asterisk (*) represents statistical significance (*p* < 0.05).
